# Serum vitamin D, intact parathyroid hormone, and Fetuin A concentrations were associated with geriatric sarcopenia and cardiac hypertrophy

**DOI:** 10.1038/srep40996

**Published:** 2017-01-23

**Authors:** Wei-Ting Chang, Chih-Hsing Wu, Ling-Wei Hsu, Po-Wei Chen, Jia-Rong Yu, Chin-Sung Chang, Wei-Chuan Tsai, Ping-Yen Liu

**Affiliations:** 1 Division of Cardiology, Department of Internal Medicine, Chi Mei Medical Center, Tainan, Taiwan; 2 Division of Cardiology, Department of Internal Medicine, College of Medicine, National Cheng Kung University Hospital, Tainan, Taiwan; 3 Department of Biotechnology, Southern Taiwan University of Science and Technology, Tainan, Taiwan; 4 Department of Family Medicine, National Cheng Kung University Hospital, Tainan, Taiwan; 5 Institute of Basic Medical Sciences, College of Medicine, National Cheng Kung University, Tainan, Taiwan; 6 Institute of Clinical Medicine, College of Medicine, National Cheng Kung University, Tainan, Taiwan; 7 Graduate Institute of Clinical Medical Sciences, College of Medicine, China Medical University, Taichung, Taiwan

## Abstract

With aging, intact parathyroid hormone (iPTH) increases. It plays a crucial role in left ventricular hypertrophy (LVH). Also, 25-hydroxy vitamin D (Vit-D) and iPTH have been observed to be determinants of muscle wasting known as sarcopenia. Fetuin A (FetA), a systemic calcification inhibitor, involves in the development of diastolic heart failure. Hence, we hypothesized that the interplay among FetA, Vit-D and iPTH may contribute to sarcopenic LVH among the elders. We analyzed a database from the Tianliao Old People study with 541 elders (≥65 years) in a Taiwan’s suburban community. After excluding patients with renal function impairment, 120/449 (26.7%) patients were diagnosed with sarcopenia. Sarcopenic patients had lower serum Vit-D levels but higher FetA as well as iPTH. Notably, sarcopenic patients with LVH had significantly lower FetA and higher iPTH levels. In multivariate logistic regression analysis, only the increase in iPTH was independently associated with sarcopenic LVH (Odds ratio: 1.05; confidence interval: 1.03–1.08, *p* = 0.005). Using iPTH >52.3 ng/l as a cutoff point, the sensitivity and specificity was 66% and 84%, respectively. In conclusion, FetA, Vit-D, and iPTH levels were all associated with sarcopenia in this geriatric population. Among them, iPTH specifically indicates patients with sarcopenic LVH.

With aging, the incidence of sarcopenia, an involuntary reduction in fatty-free muscle mass, and cardiac hypertrophy increases^1^,^2^,^3^,^4^. A growing number of studies have reported a continuous loss of myocytes subsequent to reactive hypertrophy of the remaining cells in age-related cardiac changes, or cells filled with fibrotic, adipose tissue in the extracellular matrix^4^,^5^. Although age-related cardiac changes have been well described in humans and rats, only a few studies have determined whether sarcopenia occurs in cardiac muscle^5^,^6^,^7^,^8^. Notably, the balance between vitamin D (Vit-D) and parathyroid hormone (PTH) has been considered a key regulator of muscle strength^9^. In a longitudinal aging study, low Vit-D and high PTH concentrations were concluded to be determinants of sarcopenia^10^. Increased PTH-induced cardiac hypercontractility subsequently causes reactive cardiac hypertrophy^11^. Fetuin-A (FetA) is a calcification inhibitor and is involved in insulin resistance^12^,^13^,^14^,^15^,^16^. It was a potential regulator of cardiovascular disease, and also recently reported to be a main factor in adjusting body composition remodeling in the geriatric population^17^. Also, FetA- and Vit-D receptor- knockout mice had significant ventricular hypertrophy and diastolic dysfunction^18^.

We previously reported a high concentration of FetA was associated with left ventricular dysfunction in participants with sarcopenia^19^. However, whether there is any interplay between these mineral-modification factors according to the development of heart failure in sarcopenic populations remains indeterminate. Herein, we aimed to study FetA, Vit-D, and PTH concentrations in the elderly (≥65 years old) with or without sarcopenia and left ventricular hypertrophy (LVH).

## Results

### Clinical and echocardiographic characteristics of participants with and without sarcopenia

One hundred twenty (26.7%) of the 449 participants were diagnosed with sarcopenia (Sarc^+^). Sarc^+^ participants were significantly (all *p* = 0.001) older, were more likely to be female, and had smaller waist circumferences, lower BMIs, and lower mean blood pressure (BP) (Table 1). Echocardiography showed preserved E/E′. Generally, their serologic profiles were similar, except for lower serum Vit-D concentrations (43.7 ± 11.5 vs. 41.0 ± 10.8 ng/ml, *p* = 0.04) but higher FetA (621.1 ± 140.7 vs. 697.3 ± 179.6 μg/dl, *p* < 0.001) and PTH (44.1 ± 28.6 vs. 52.1 ± 29.1 ng/l, *p* = 0.01) concentrations. None had specific heart failure symptoms. LVH^+^ participants (75/449, 16.7%) had larger waist circumferences, higher systolic blood pressure (SBP), and higher serum PTH concentrations (Supplementary Table 1). Their serum calcium, phosphate, Vit-D, and FetA concentrations were not significantly different from those of LVH^−^ participants.

In this case control study, 10 (8.3%) of the 120 Sarc^+^ participants were also LVH^+^ (Table 2). Significantly (*p* < 0.001) more participants diagnosed as Sarc^+^ LVH^+^ were male and had relatively lower SBP than did Sarc^+^ LVH^−^ participants, their prevalence of stroke, coronary artery disease (CAD), and arrhythmia was not significantly different. Notably, Sarc^+^ LVH^+^ participants had significantly lower FetA concentrations (696.9 ± 171.9 vs. 547.6 ± 142.6 μg/dl, *p* < 0.03) but significantly higher PTH concentrations (44.7 ± 17.6 vs. 63.7 ± 17.9 ng/l, *p* = 0.007) no significant differences in Vit-D, calcium, or phosphate concentrations. Echocardiography showed significantly higher LVMI and a lower E/E′ in the Sarc^+^ LVH^+^ subgroup. The incidence of diastolic dysfunction (impaired relaxation [E′ < 0.08 m/s] and high left ventricular end-diastolic pressure [E/E′ > 8]) was significantly higher in the Sarc^+^ LVH^+^ subgroup than in the other subgroups.

There were more Sarc^+^ and Sarc^+^ LVH^+^ female participants in the fourth quartile of PTH (concentration > 51.82 ng/l) (Supplementary Table 2). Likewise, there were significantly more Sarc^+^ LVH^+^ female participants in the first quartile of Vit-D (Supplementary Table 3).

### The diagnostic effect of PTH on Sarc^+^ LVH^+^ participants

In the multivariate logistic regression, female gender, a lower BMI, and a lower Vit-D concentration were significantly correlated with sarcopenia (Supplementary Table 4), but only a higher PTH concentration significantly predicted that a participant was Sarc^+^ LVH^+^ (odds ratio: 1.05, 95% CI: 1.03–1.08, *p* < 0.005) (Table 3).

**Figure 1:**
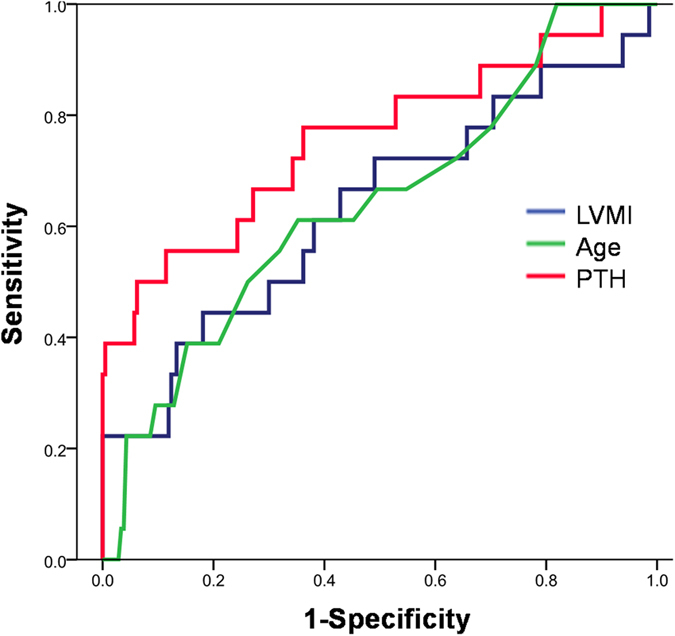
The areas under the ROC curves for the predictor of sarcopenic left ventricular hypertrophy were 0.7 for left ventricular mass index (LVMI), 0.67 for age and 0.75 for parathyroid hormone (PTH).

The area under the ROC curve was 0.75 for Sarc^+^ LVH^+^ compared with 0.7 for LVMI and 0.67 for age (Fig. 1). The ROC curve, using PTH > 52.3 ng/l as a cutoff point, showed a sensitivity of 66% and a specificity of 84% for a Sarc^+^ LVH^+^ diagnosis.

## Discussion

The present study’s three main findings are: (1) sarcopenia and cardiac hypertrophy were not rare in the geriatric population; (2) PTH was significantly associated with Sarc^+^ LVH^+^, independently of hypertension and serum calcium and phosphate concentrations; (3) PTH >52.3 ng/l is a biomarker that indicates Sarc^+^ LVH^+^.

Aging is a major cause of congestive heart failure^5^,^6^. Clinically, 40–50% percent of patients with symptomatic heart failure have preserved systolic function, which is called “diastolic dysfunction”^5^,^8^. People with diastolic heart failure tend to be older and have sarcopenia, chronic heart failure, and cardiac hypertrophy^8^. Physiologically, diastolic dysfunction occurs when a rigid ventricle fails to properly fill^7^,^8^. Importantly, the survival of participants with diastolic dysfunction is similar to that of patients with systolic dysfunction^5^. The importance of investigating diastolic heart failure has recently been emphasized. One study^20^ reported that in Vit-D receptor-knockout mice, suppressing the pro-hypertrophic calcineurin pathway led to cardiac hypertrophy^20^, and another^21^ reported that PTH activates the protein kinase C of cardiomyocytes and induces hypertrophic growth. PTH treatment, though being shown to improve cardiac function after myocardial infarction by increasing neovascularization and cell survival, also increases fibrosis, which accelerates decompensated heart failure^22^. Notably, the combination of lower Vit-D and higher PTH concentrations appears to be independently associated with sudden cardiac death in older adults without overt cardiovascular disease^23^. FetA, although valued as a negative acute-phase protein, has anti-inflammatory characteristics and protects patients with renal failure from cardiovascular events. Emerging evidence indicated its dark sides in insulin resistance, myocardial infarction and ischemic stroke^24^,^25^. Despite causing cardiac hypertrophy and diastolic dysfunction in FetA-knockout mice^18^, the actual role of FetA in the development of heart failure remains uncertain.

Other studies^3^,^4^ have hypothesized several mechanisms for sarcopenia. Some hypothesize sarcopenic obesity: the notion that adipose tissue replaces lost myocytes^3^. A recent study^17^ showed that higher FetA concentrations were associated with the accumulation of visceral fat tissue in well-functioning, community-living older people, which corresponds to the spirit of sarcopenic obesity. Vit-D directly affected various aspects of muscle function, including myofibrillar protein degradation and impaired calcium transmission in the mitochondria^26^. One study^27^ reported that a Vit-D deficiency in the elderly causes secondary hyperparathyroidism. The coexistence of low Vit-D and high PTH has been a determinant of muscle strength loss in the elderly^10^.

In our previous study of the TOP population^19^, the Sarc^+^ group tended to have a higher concentration of FetA, which was associated with poor heart function. More recently, we documented^28^ that the hearts of the elderly were stiffer than those of younger people. In a mouse model that investigated cardiac sarcopenia^7^, senescent mice showed both intracellular lipid accumulation and a decrease in glycogen storage. In humans, many mechanisms have been proposed for the aging process, including mitochondrial function changes, senescence adaption, and telomerase shortening^29^,^30^. Quantitative studies^7^,^31^ have shown that a continuous loss of myocytes is accompanied by reactive hypertrophy of the remaining cells, or that the extracellular matrix is filled with fibrotic, adipose tissue. The adaptation of the left ventricle is a complex process of hormonal, structural, and hemodynamic factors, and most serological markers changed with aging^7^,^31^. In the present study, we analyzed the related mineralizing serological markers—FetA, Vit-D, and PTH—in elderly Sarc^+^ LVH^+^, Sarc^+^ LVH^−^, Sarc^−^ LVH^+^, and Sarc^−^ LVH^−^ participants. To prevent the disturbance of the calcium and phosphate balance in participants with impaired renal function, we focused only on participants with preserved renal function. Consequently, in addition to low Vit-D concentrations in Sarc^+^ participants, we discovered the specific association between PTH and Sarc^+^ LVH^+^ participants independently of hypertension and of calcium or phosphate concentration.

Our study has some limitations. First, only 120 of our 449 (26.7%) participants presented as Sarc^+^ LVH^+^, and the statistical findings might be over-fitted. Second, cardiac dysfunction and hypertrophy were defined based on echocardiography, and none of participants diagnosed with these diseases presented with clinical symptoms. Third, through this case control study, identifying the causal relationship between iPTH and diastolic dysfunction faces difficulty. More studies in the future to correlate the echocardiographic findings to clinical symptoms are required.

## Conclusion

The mineralizing markers FetA, Vit-D, and PTH were all associated with sarcopenia in our participants. PTH identified Sarc^+^ LVH^+^ participants. However, more investigation and longitudinal follow-ups are required to verify the clinical effects of Sarc^+^ LVH^+^.

## Methods

### Study Population

Tianliao Township is a suburban community of Kaohsiung City, Taiwan. Nearly a quarter (23.7%) of its 31,000 residents are >65 years old. The Tianliao Old People (TOP) study^19^ has continuously been conducted for the elders since 2009 in order to develop better community-oriented primary-care services. Based on the 2010 census, there were 1,033 elderly men in Tianliao. In July 2010, we used the whole community sampling method for an epidemiological survey. After excluding 269 empty houses, 21 deaths, and 62 non-ambulatory residents, only 681 residents were eligible. The response rate was 60.8% and the statistical power was 0.80. After residents with left ventricular systolic dysfunction (left ventricular ejection fraction <50%) and renal function impairment (estimated glomerular filtration rate [eGFR] <60 mL/min per 1.73 m^2^, based on the Kidney Disease: Improving Global Outcomes (KDIGO) recommendation <http://kdigo.org/home/>), and residents taking calcium, PTH, or Vit-D supplements had been excluded, 541 residents were enrolled. Based on the medical history reported by each participant, medical information about strokes, coronary artery disease, arrhythmia, and cancer was recorded. Anthropometric (e.g., body mass index [BMI]) and duplicate waist circumference measurements were done on bare skin midway between the lower rib margin and the iliac crest at the end of normal expiration. Each participant signed an informed consent before they were examined. This study was approved by the Institutional Review Board of National Cheng Kung University Hospital (IRB: ER-99-111) and all methods were performed in accordance with our local guidelines and clinical regulations.

### Laboratory Methods

After the participants had fasted for at least 8 hours, peripheral blood samples were collected and centrifuged at 3,000 rpm for 15 minutes at 4 °C. The samples were frozen and sent to a central laboratory for analysis. Serum PTH, Vit-D, and FetA were determined using an ELISA assay (Biovendor Laboratory Medicine, Brno, Czech Republic). The minimum detectable concentrations of PTH, Vit-D, and FetA were 0.2, 0.8, and 9.38 ng/ml, respectively.

### Definition of Sarcopenia

To define sarcopenia in this study, we measured skeletal muscle mass (SMM) using a bioelectrical impedance analyzer (BioScan 920; Maltron, Rayleigh, Essex, UK), a convenient, noninvasive, and radioisotope-free tool with an operating frequency of 50 kHz at 800 mA. Participants were supine on a non-conductive surface with their arms abducted away from their trunk and their legs slightly separated for 5 minutes. Four electrodes and cables were attached to their right hand and ankle. When the measurements stabilized, the analyzer displayed resistance directly and immediately. SMM was calculated using the BIA equation (described elsewhere)^2^:


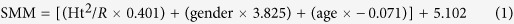



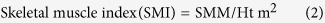


The associated units were: height in cm; resistance in ohms (Ω); gender (51 men, 50 women); and age in years. Absolute SMM was converted to an SMI by dividing it by height in square meters (SMM/Ht m^2^). Based on the literature^1^,^2^,^19^, the normal range of the SMI was defined as >7.23 for men and >5.67 for women.

Sarcopenia was defined based on the 2010 criteria of the European Working Group on Sarcopenia in Older People (EWGSOP)^2^ as presenting 3 of the following components: (1) loss of muscle mass: an SMI of ≥2 standard deviations (SDs) less than the normal gender-specific mean. In the studied population, an SMI <7.23 in men and <5.67 in women meant being assigned to the sarcopenic group; (2) low muscle strength: the maximal weight of hand-grasping <30 pounds in men and <20 pounds in women; (3) low physical performance: a maximal walking speed <0.8 m/s.

### Echocardiography

Standard echocardiography was done (Vivid I; GE Vingmed Ultrasound AS, Horten, Norway) using a 3.5-MHz multiphase-array probe, based on the recommendations of the American Society of Echocardiography^32^. The chamber dimensions, left ventricular (LV) mass, LV ejection fraction (LVEF), and left ventricular mass index (LVMI) were all determined using 2D and 3D M-mode methods.


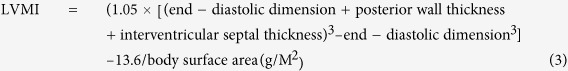


LVMIs >102 g/M^2^ in men and >88 g/M^2^ in women were defined as cardiac hypertrophy. Transmitral Doppler flow velocity was obtained from an apical four-chamber view, and peak early mitral inflow velocity (E), peak atrial velocity (A), and the E/A ratio were recorded. Early mitral inflow velocity and early annular diastolic velocity (E′) were also measured to estimate the LV end-diastolic pressure (E/E′). Both the medial and lateral early annular diastolic velocity values were measured to determine the mean value. Echocardiographic diastolic dysfunction was defined as the coexistence of impaired relaxation (E′ < 0.08 m/s [determined using tissue Doppler]) and elevated mean LV end-diastolic pressure (E/E′ > 8)^23^. To distinguish them from the population with preserved LV systolic function, participants with an LVEF <50% were defined as having an impaired LV systolic function (or left ventricular dysfunction [LVD])^24^. The systolic and diastolic function of each echocardiographic image was analyzed in triplicate by experienced cardiologists. Based on the criteria of sarcopenia and of LVH, participants were assigned to sarcopenia^Positive^ LVH^Negative^ (Sarc^+^-LVH^−^), Sarc^−^-LVH^+^, Sarc^−^-LVH^−^, and Sarc^+^-LVH^+^ groups.

### Statistical Analysis

Data management and statistical analyses were done using SPSS 18.0. Continuous variables with a normal distribution are expressed as means ± standard deviation (SD). Other continuous variables with a non-normal distribution are expressed as means ± standard error (SE). Continuous variables were compared using Student’s *t* test for normally distributed values. To validate the association between sarcopenia and heart function, we separated the participants based on whether they had sarcopenia, LVH, or both. Group differences were analyzed using analysis of variance (ANOVA). Significant differences verified using a Tukey post hoc test in analysis were entered into multivariate analysis. Odds ratios (ORs) and 95% confidence intervals (CIs) were assessed using logistic regression. Statistical tests were 2-sided; significance was set at *p* < 0.05. Receiver operating characteristic (ROC) curve analysis was used to determine the optimal cutoff values of PTH in participants with and without sarcopenic cardiomyopathy. The best cutoff value was defined as the point with the highest sum of sensitivity and specificity.

## Additional Information

**How to cite this article**: Chang, W.-T. *et al*. Serum vitamin D, intact parathyroid hormone, and Fetuin A concentrations were associated with geriatric sarcopenia and cardiac hypertrophy. *Sci. Rep.* **7**, 40996; doi: 10.1038/srep40996 (2017).

**Publisher's note:** Springer Nature remains neutral with regard to jurisdictional claims in published maps and institutional affiliations.

## Supplementary Material

Supplementary Tables

## Figures and Tables

**Table 1:** Clinical and echocardiographic characteristics: comparison between Sarc^−^ and Sarc^+^ groups.

	Without Sarcopenia (Sarc^−^)	With Sarcopenia (Sarc^+^)	*p*
	(329/449, 73.3%)	(120/449, 26.7%)	
Age (years)	75.20 ± 5.8	80.00 ± 6.1	<0.001
Male (%)	190.00 (57.7)	33 (27.5)	<0.001
Waist circumference (cm)	88.40 ± 9.7	78.70 ± 9	<0.001
BMI (kg/m^2^)	25.10 ± 3.8	21.40 ± 2.9	<0.001
SBP (mmHg)	135.70 ± 20.9	127.40 ± 19.9	0.002
DBP (mmHg)	77.60 ± 11.9	72.70 ± 10.9	0.001
Medical History (%)			
Previous stroke	19 (5.7)	4 (3.3)	0.90
CAD	36 (10.9)	8 (6.6)	0.85
Arrhythmia	10 (3)	3 (2.5)	0.33
Cancer	9 (2.7)	6 (5)	0.65
Serology profiles			
eGFR	83.20 ± 16.2	71.00 ± 8.9	0.53
HbA_1_c (%)	6.10 ± 0.9	6.10 ± 1.2	0.92
TG (mg/dl)	130.40 ± 80.1	118.50 ± 61.3	0.24
Cholesterol (mg/dl)	201.80 ± 36.8	204.90 ± 34.9	0.51
Calcium (mg/dl)	9.10 ± 0.4	9.10 ± 0.4	0.61
Phosphate (mg/dl)	3.50 ± 0.5	3.60 ± 0.4	0.36
FetA (μg/dl)	621.10 ± 140.7	697.30 ± 179.6	<0.001
Vit-D (ng/ml)	43.70 ± 11.5	40.90 ± 10.8	0.02
PTH (ng/l)	44.10 ± 28.6	52.10 ± 29.1	0.01
Echocardiographic parameters			
LVMI (g/M^2^)	82.10 ± 19.9	77.10 ± 16.8	0.34
LVEF (%)	71.30 ± 6.4	70.00 ± 4.7	0.34
E/A	0.70 ± 0.2	0.70 ± 0.3	0.69
E′ (m/s)	0.08 ± 0.02	0.08 ± 0.02	0.61
E/E′	7.60 ± 2.1	9.20 ± 4.4	0.002
Diastolic dysfunction	50 (15.2)	18 (15)	0.06

Data are expressed as n (%) or mean ± standard error. BMI = body mass index; SBP = systolic blood pressure; DBP = diastolic blood pressure; eGFR = estimated glomerular filtration rate; CAD = coronary artery disease; HbA_1_C = glycated hemoglobin; TG = triglyceride; FetA = Fetuin A; Vit-D = 25-hydroxy vitamin D; PTH = parathyroid hormone; LVMI = left ventricular mass index; LVEF = left ventricular ejection fraction; E/A = transmitral valve E to A velocity ratio; E/E′ = mitral early filling velocity to early diastolic mitral annular velocity ratio.

**Table 2:** Clinical and echocardiographic characteristics: comparison between Without Sarcopenia and Without Left Ventricular Hypertrophy (Sarc^−^ LVH^−^), Without Sarcopenia and With Left Ventricular Hypertrophy (Sarc^−^ LVH^+^), With Sarcopenia and Without Left Ventricular Hypertrophy (Sarc^+^ LVH^−^), With Sarcopenia and With Left Ventricular Hypertrophy (Sarc^+^ LVH^+^) groups.

	Without Sarcopenia (Sarc^−^)		With Sarcopenia (Sarc^+^)		*F*	*p*
	(329/449, 73.3%)		(120/449, 26.7%)			
	Sarc^−^ LVH^−^	Sarc^−^ LVH^+^	Sarc^+^ LVH^−^	Sarc^+^ LVH^+^		
	(n = 272, 60.57%)	(n = 57, 12.69%)	(n = 110, 24.49%)	(n = 10, 2.2%)		
Age (years)	75.80 ± 5.9	77.70 ± 6.1^a^	80.20 ± 6.1^a^	77.50 ± 7.7	6.3	<0.001
Male (%)	158 (58)	37 (64.9)	24 (21.8)^a,b^	4 (40)^a,b^		<0.001
WC (cm)	87.90 ± 9.6	91.95 ± 8.49	78.81 ± 9.22^a,b^	78.80 ± 1.83^a,b^	15.7	<0.001
BMI (kg/m^2^)	24.40 ± 4	25.82 ± 2.9	21.50 ± 3^a^	20.00 ± 0.1^a,b^	11.5	<0.001
SBP (mmHg)	133.90 ± 19.4	143.90 ± 23.2	128.20 ± 19.9^a^	120.00 ± 14.1^a,b^	4.6	0.001
DBP (mmHg)	76.90 ± 11.7	80.40 ± 14.1	78.90 ± 10.6	80.50 ± 9.2^a^	3.9	0.004
Previous stroke	16 (5.8)	3 (5.2)	3 (2.7)	1 (10)		0.33
CAD	28 (10.3)	8 (14)	6 (5.4)	2 (20)		0.06
Arrhythmia	9 (3.2)	1 (1.7)	2 (1.8)	1 (10)		0.08
Cancer	5 (1.8)	4 (7)	5 (4.5)	1 (10)		0.34
GFR	80.50 ± 12.5	83.90 ± 11.3	78.20 ± 8.9	75.10 ± 9.8	3.2	0.21
HbA1c (%)	6.02 ± 0.9	5.95 ± 0.58	6.10 ± 1.2	6.40 ± 0.9	1.3	0.282
TG (mg/dl)	119.20 ± 76.5	109.30 ± 51.1	120.00 ± 61.9	95.00 ± 46.7	0.3	0.902
Cholesterol (mg/dl)	195.10 ± 35.3	193.80 ± 30.3	206.00 ± 34.6	158.00 ± 28.3	2.1	0.082
Cholesterol (mg/dl)	9.10 ± 0.3	8.80 ± 0.6	8.90 ± 0.3	8.80 ± 0.5	0.8	0.15
Phosphate (mg/dl)	3.40 ± 0.8	3.70 ± 1.7	3.50 ± 0.5	3.60 ± 0.8	0.7	0.51
FetA (μg/dl)	613.60 ± 136.7	602.10 ± 88.6	696.90 ± 171.9	547.60 ± 142.5^a,b,c^	2.8	0.026
Vit-D (ng/ml)	48.30 ± 10.4	47.20 ± 9.8	40.20 ± 10.8	40.30 ± 6.2	5.4	0.08
PTH (ng/l)	38.40 ± 12.7	38.30 ± 14.9	44.70 ± 17.6	63.70 ± 17.9^a,b^	3.6	0.007
LVMI (g/M2)	75.30 ± 13.9	113.50 ± 11.9^a^	75.10 ± 12.1	103.60 ± 1.5^a,c^	58.1	<0.001
LVEF (%)	71.30 ± 6.2	71.10 ± 6.6	69.90 ± 4.9	70.50 ± 0.7	0.2	0.924
E/A	0.70 ± 0.2	0.70 ± 0.2	0.70 ± 0.2	0.70 ± 0.2	7.3	0.1
E′ (m/s)	0.08 ± 0.01	0.08 ± 0.02	0.09 ± 0.01	0.06 ± 0.02	2.1	0.089
E/E′	7.50 ± 2	7.90 ± 2.3	7.80 ± 1.3	9.60 ± 1.6	3.4	0.08
Diastolic dysfunction	29 (10.7)	21 (36.8)	8 (7.2)	10 (100)^a,b,c^	8.9	0.001

Data are n (%) or mean ± standard error. Abbreviations presented in Table 1.

^a^*P* < 0.05, compared with Sarc^−^ LVH^−^.

^b^*P* < 0.05, compared with Sarc^−^ LVH^+^.

^c^p < 0.05, compared with values of Sarc^−^ LVH^+^.

**Table 3:** Univariate and multivariate regression analyses to identify participants with sarcopenic left ventricular hypertrophy (Sarc^+^ LVH^+^).

	Univariate Analysis		Multivariate Analysis	
	OR (95% CI)	*p*	OR (95% CI)	*p*
Age (years)	1.07 (1.01–1.14)	0.02		
Male (%)	0.85 (0.37–1.96)	0.7		
FetA (μg/dl)	1.02 (1.00–1.08)	0.01	1.01 (0.999–1.020)	0.06
PTH (ng/l)	1.80 (1.2–2.7)	<0.001	1.05 (1.03–1.08)	<0.001
LVMI (g/M2)	1.56 (1.08–1.84)	0.01	1.01 (0.85–1.32)	0.12

Data are expressed as n (%) or mean ± standard error. OR = odds ratio; CI = confidence interval; FetA = Fetuin A; PTH = parathyroid hormone; LVMI = left ventricular mass index.
