# Supplementary material for: Serum vitamin D, intact parathyroid hormone, and Fetuin A concentrations were associated with geriatric sarcopenia and cardiac hypertrophy
Source: Sci Rep. 2017 Jan 23;7:40996. doi: 10.1038/srep40996 (PMC5253676; doi:10.1038/srep40996)
Supplement: Supplementary Tables [file srep40996-s1.doc]

**Serum vitamin D, intact parathyroid hormone, and Fetuin A concentrations were associated with geriatric sarcopenia and cardiac hypertrophy**

Wei-Ting Chang, Chih-Hsing Wu, Ling-Wei Hsu, Po-Wei Chen, Jia-Rong Yu, Chin-Sung Chang, Wei-Chuan Tsai, and Ping-Yen Liu

**Supplementary Table 1. Clinical and echocardiographic characteristics: comparison between LVH^‒^ and LVH^+^** groups

|  | LVH**^‒^** | LVH**^+^** |  |
| --- | --- | --- | --- |
|  | (n = 382/449, 85.1%) | (n = 67/449, 14.9%) | *p* |
| Age (years) | 76.40 ± 6.3 | 77.70 ± 6.1 | 0.27 |
| Male (%) | 198 (52.9) | 31 (41.3) | 0.05 |
| WC (cm) | 87.10 ± 10.1 | 91.20 ± 8.8 | 0.03 |
| BMI (kg/m^2^) | 24.10 ± 4.1 | 25.50 ± 3.1 | 0.05 |
| SBP (mmHg) | 133.20 ± 19.5 | 142.60 ± 23.4 | 0.01 |
| DBP (mmHg) | 76.50 ± 11.8 | 80.40 ± 13.8 | 0.08 |
| Medical History (%) | | | |
| Previous stroke | 19 (4.9) | 4 (5.9) | 0.08 |
| CAD | 34 (8.9) | 10 (14.9) | 0.82 |
| Arrhythmia | 11 (2.9) | 2 (2.9) | 0.55 |
| Cancer | 10 (2.6) | 5 (7.4) | 0.05 |
| Serology profiles | | | |
| GFR | 76.20 ± 12.8 | 73.90 ± 10.8 | 0.7 |
| HbA_1_c (%) | 6.00 ± 0.9 | 5.90 ± 0.6 | 0.71 |
| Calcium (mg/dl) | 9.10 ± 0.4 | 8.90 ± 0.5 | 0.08 |
| Phosphate (mg/dl) | 3.50 ± 0.5 | 3.70 ± 0.8 | 0.16 |
| FetA (μg/dl) | 614.30 ± 148.2 | 590.30 ± 100.4 | 0.37 |
| Vit-D (ng/ml) | 48.30 ± 10.9 | 46.50 ± 10.3 | 0.38 |
| PTH (ng/l) | 43.20 ± 29.9 | 51.80 ± 65.5 | 0.02 |
| Echocardiographic parameters | | | |
| LVMI (g/M^2^) | 75.10 ± 13.9 | 112.90 ± 11.8 | < 0.001 |
| LVEF (%) | 71.30 ± 6.2 | 71.00 ± 6.4 | 0.78 |
| E/A | 0.70 ± 0.2 | 0.70 ± 0.2 | 0.49 |
| E′ (m/s) | 0.08 ± 0.01 | 0.08 ± 0.02 | 0.12 |
| E/E′ | 7.60 ± 2.2 | 7.90 ± 2.3 | 0.5 |
| Diastolic dysfunction | 37 (9.6) | 22 (32.8) | 0.02 |

Data are expressed as n (%) or mean ± standard error. Abbreviations presented in Table 1.

**Supplementary Table 2.** The clinical and echocardiographic characteristics of participants categorized by quartiles of PTH

|  | 14.5≤Q1<31.6 | 31.6≤Q1<39.3 | 39.3≤Q1<51.8 | 51.8≤Q1≤411 | *F* | *p* |
| --- | --- | --- | --- | --- | --- | --- |
| Age (years) | 75.3 ± 6.3 | 75.7 ± 6.6 | 75.9 ± 5.3 | 76.7 ± 6.5 | 1.238 | 0.29 |
| Male (%) | 71 (60.2) | 78 (54.2) | 70 (53.4) | 59 (41.3) |  | 0.01 |
| WC (cm) | 86.7 ± 10.2 | 87.5 ± 9.2 | 86.8 ± 10.7 | 87.2 ± 10.9 | 0.172 | 0.91 |
| BMI (kg/m^2^) | 24.2 ± 3.8 | 24.7 ± 3.2 | 24.8 ± 3.8 | 25.2 ± 4.9 | 1.164 | 0.32 |
| SBP (mmHg) | 133.7 ± 20.8 | 131 ± 17.5 | 135.8 ± 21.9 | 138 ± 22.2 | 3.006 | 0.08 |
| DBP (mmHg) | 75.8 ± 11.9 | 75.5 ± 10.7 | 77.5 ± 12.2 | 79.7 ± 11.8 | 3.802 | 0.12 |
| Sarc**^+^** | 15 (10.7) | 16 (11.4) | 18 (12.8) | 37 (26.4) |  | 0.002 |
| Sarc**^+^** LVH**^+^** | 1 (0.7) | 3 (2.1) | 4 (2.8) | 15 (10.7) |  | < 0.001 |
| FetA (μg/dl) | 613.7 ± 149.7 | 644.4 ± 147.6 | 627.5 ± 143.2 | 645.9 ± 163.1 | 1.193 | 0.31 |
| LVMI (g/M^2^) | 81.8 ± 20.9 | 80.7 ± 18.4 | 79.1 ± 19.5 | 86.5 ± 26.4 | 1.158 | 0.32 |
| LVEF (%) | 69.3 ± 8.1 | 67.7 ± 8.6 | 68.7 ± 9.6 | 65.9 ± 8.6 | 2.269 | 0.08 |
| E/A | 0.7 ± 0.2 | 0.7 ± 0.2 | 0.7 ± 0.2 | 0.7 ± 0.2 | 0.664 | 0.57 |
| E′ (m/s) | 0.08 ± 0.02 | 0.08 ± 0.02 | 0.09 ± 0.02 | 0.08 ± 0.02 | 0.664 | 0.57 |
| E/E′ | 7.7 ± 2.6 | 7.6 ± 2.5 | 7.5 ± 2.2 | 8.6 ± 3 | 0.664 | 0.57 |
| Diastolic | 18 (23.1) | 21 (23.6) | 14 (19.7) | 24 (33.3) |  | 0.26 |
| dysfunction |  |  |  |  |  |  |

Abbreviations presented in Table 1.

**Supplementary Table 3.** The clinical and echocardiographic characteristics of participants categorized by quartiles of Vit-D

|  | 10.4 ≤Q1<35.2 | 35.2≤Q1<42.2 | 42.2≤Q1<51.9 | 51.9≤Q1≤70 | *F* | *p* |
| --- | --- | --- | --- | --- | --- | --- |
| Age (years) | 75.6 ± 6.1 | 75.2 ± 5.7 | 76.6 ± 6.7 | 76.4 ± 6.3 | 1.14 | 0.33 |
| Male (%) | 35 (29.5) | 53 (41.1) | 75 (56.4) | 109 (77.3) |  | < 0.001 |
| WC (cm) | 86.1 ± 9.1 | 87.7 ± 10.2 | 88.1 ± 11 | 86.5 ± 10.6 | 1.15 | 0.33 |
| BMI (kg/m^2^) | 24.9 ± 3.4 | 24.9 ± 3.9 | 25.2 ± 5 | 24 ± 3.5 | 2.02 | 0.09 |
| SBP (mmHg) | 135.2 ± 19 | 138 ± 21.9 | 132.9 ± 20.1 | 132.3 ± 21.5 | 1.57 | 0.18 |
| DBP (mmHg) | 76.7 ± 10.8 | 78.4 ± 12.4 | 77.1 ± 12 | 76.6 ± 11.8 | 0.49 | 0.74 |
| Sarcopenia | 25 (17.8) | 22 (15.7) | 19 (13.5) | 20 (14.3) |  | < 0.001 |
| SLVH | 2 (1.4) | 3 (2.1) | 3 (2.1) | 2 (1.4) |  | 0.58 |
| FetA (μg/dl) | 658.6 ± 169.4 | 641.5 ± 149.5 | 620.3 ± 131.7 | 615.6 ± 152.8 | 1.58 | 0.18 |
| LVMI (g/M^2^) | 84.3 ± 27.8 | 82.7 ± 19.2 | 81.2 ± 17 | 80.8 ± 22.3 | 0.25 | 0.86 |
| LVEF (%) | 65.3 ± 10.3 | 68 ± 8 | 68.2 ± 8.4 | 69 ± 8.2 | 2.47 | 0.06 |
| E/A | 0.7 ± 0.2 | 0.7 ± 0.2 | 0.7 ± 0.2 | 0.7 ± 0.2 | 0.44 | 0.72 |
| E′ (m/s) | 0.09 ± 0.02 | 0.08 ± 0.01 | 0.08 ± 0.02 | 0.08 ± 0.02 | 0.53 | 0.66 |
| E/E′ | 8.6 ± 3.6 | 8.2 ± 2.7 | 7.6 ± 2.4 | 7.3 ± 1.9 | 4.12 | 0.007 |
| Diastolic | 16 (28.6) | 20 (28.2) | 17 (21) | 23 (23) |  | 0.64 |
| dysfunction |  |  |  |  |  |  |

Abbreviations presented in Table 1

**Supplementary Table 4. Univariate and multivariate regression analyses to identify Sarc^+^ participants**

|  | Univariate Analysis | | Multivariate Analysis | |
| --- | --- | --- | --- | --- |
|  | OR (95% CI) | *p* | OR (95% CI) | *p* |
| Age (years) | 1.07 (1.01-1.14) | 0.02 |  |  |
| Male (%) | 0.71 (0.64-0.82) | 0.001 | 0.98 (0.72-2.43) | 0.03 |
| BMI (kg/M2) | 0.65 (0.59-0.71) | 0.001 | 0.43 (0.29-0.62) | 0.01 |
| WC (cm) | 0.89 (0.87-0.91) | 0.001 | 0.95 (0.87-0.97) | 0.07 |
| FetA (μg/dl) | 1 (0.99-1.03) | 0.03 |  |  |
| PTH (ng/l) | 1 (0.99-1.02) | 0.06 |  |  |
| Vit-D (ng/ml) | 0.73 (0.69-0.94) | 0.001 | 0.90 (0.78-0.97) | 0.01 |

Abbreviations presented in Table 1
